# Efficacy of acupuncture for motor dysfunction in early Parkinson’s disease: protocol for a randomized, single-blind, sham-controlled clinical trial

**DOI:** 10.3389/fmed.2025.1699907

**Published:** 2025-11-19

**Authors:** Qifu Li, Yuquan Shen, Yangyang Wang, Junxia Liu, Ziwen Chen, Weiming Luo, Yan Chen, Yunnan Liu, Xinghua Zhu, Simin Xue, Yanru Chen, Dehua Li, Taipin Guo, Jun Zhou, Fanrong Liang

**Affiliations:** 1 College of Acupuncture and Tuina, Chengdu University of Traditional Chinese Medicine, Kunming, China; 2 The Neurology Department, West China Longquan Hospital Sichuan University/The First People’s Hospital of Longquanyi District, Chengdu, China; 3 Department of Acupuncture, The Hospital of Chengdu University of Traditional Chinese Medicine, Chengdu, Sichuan, China; 4 School of Second Clinical Medicine/The Second Affiliated Hospital, Yunnan University of Chinese Medicine, Kunming, China; 5 Key Laboratory of Acupuncture for Senile Disease (Chengdu University of TCM), Ministry of Education, Chengdu, China

**Keywords:** Parkinson’s disease, acupuncture, composite clinical motor, long-term follow-up, randomized controlled trial, study protocol

## Abstract

**Introduction:**

Motor dysfunction, including bradykinesia, rigidity, tremor, and gait disturbance, is the core clinical feature of Parkinson’s disease (PD) and a major cause of disability. In early-stage PD (Hoehn and Yahr ≤2.5), neurodegeneration is relatively mild, and timely intervention may help preserve motor function and delay progression. Although dopaminergic medications effectively alleviate motor symptoms, their long-term use is associated with motor fluctuations and levodopa-induced dyskinesia. Acupuncture, a low-risk, well-tolerated intervention, has shown potential in improving motor performance in PD, but high-quality evidence in early-stage patients is lacking.

**Methods and analysis:**

This single-blind, sham-controlled randomized clinical trial will enroll 120 patients with early-stage PD (disease duration ≤3 years). Participants will be randomly assigned in a 1:1 ratio (*n* = 60 per group) to the true acupuncture (TA) or sham acupuncture (SA) group using stratified block randomization (block size 4) by study center. Treatment will be administered three times weekly for 12 weeks, with follow-up to 15 months. The primary outcome is the change in composite clinical motor score (CCMS) from baseline to week 12. The secondary outcomes include Movement Disorder Society unified Parkinson’s disease rating scale (MDS-UPDRS), Purdue Pegboard Test score (PPTS), Timed Up and Go Test (TUG), 6-min walk test (6MWT), Non-Motor Symptoms Scale (NMSS), 39-item Parkinson’s Disease Questionnaire (PDQ-39), Clinical Global Impression Scale (CGI), and levodopa equivalent daily dose (LEDD). Acupuncturists will not be blinded while participants, outcome assessors, and data analysts remain blinded. The primary analysis will use intention-to-treat (ITT) with mixed-effects models for longitudinal changes, handling missing data via multiple imputation by chained equations.

**Discussion:**

This study aimed to evaluate the efficacy and safety of acupuncture in improving motor function in early-stage PD. The results will help determine its potential role as an adjunctive therapy to preserve motor function.

**Clinical trial registration:**

https://itmctr.ccebtcm.org.cn/, identifier ITMCTR2025001290.

## Introduction

Parkinson’s disease (PD) is a progressive neurodegenerative disorder characterized primarily by motor dysfunction, including bradykinesia, rigidity, tremor, and postural instability, which collectively lead to progressive disability and reduced quality of life (QoL) (1). The global burden of PD is rising rapidly with population aging, affecting over 10 million people worldwide in 2020 and projected to reach approximately 17 million by 2040 (2, 3). In China, the number of patients is expected to exceed 5 million by 2030 (4), with annual new cases approaching 880,000 by 2044 (5), imposing substantial challenges on patients, families, and the healthcare system.

Early-stage PD—commonly defined as Hoehn and Yahr (H&Y) stage ≤2.5 and disease duration ≤3 years—represents a critical therapeutic window in which neurodegeneration is relatively mild and motor function can be preserved with timely intervention (6–8). Maintaining motor performance during this stage is essential, as early declines in gait, dexterity, and coordination strongly predict long-term disability (9). Dopaminergic medications, including levodopa, dopamine agonists, and monoamine oxidase-B inhibitors, remain the mainstay of motor symptom management (10). While these agents provide short-term symptomatic relief, long-term use is associated with motor fluctuations, levodopa-induced dyskinesia, and diminishing efficacy (11, 12). Moreover, pharmacological therapy alone does not halt disease progression, underscoring the need for adjunctive interventions that can sustain motor function and potentially delay the onset of treatment-related complications.

Acupuncture, an integral component of traditional Chinese medicine (TCM), is considered safe, low-cost, and well-tolerated, with a history spanning over 3,000 years in treating tremor-related disorders in China (13). Recent randomized controlled trials and meta-analyses have demonstrated that acupuncture, electroacupuncture, and scalp acupuncture significantly improve motor symptoms in PD patients (14–16). Targeted stimulation of specific acupoints (e.g., GB34 and GV16) enhances tremor control, balance, and gait performance (14, 17), while scalp acupuncture modulates cortical activity in the middle frontal gyrus and cerebellum to improve step length and walking speed (17). Mechanistically, acupuncture modulates multiple neurobiological pathways relevant to PD. In 6-hydroxydopamine (6-OHDA) rat models, 100-Hz electroacupuncture at SP6, GB34, and ST36 restores dopamine D1 receptor expression, inhibits D2 receptor overactivation, and improves motor function by regulating dopaminergic transmission (18). It also enhances neurotrophic signaling by upregulating brain-derived neurotrophic factor (BDNF) and glial cell line-derived neurotrophic factor (GDNF), promoting dopaminergic neuron survival (19, 20). Furthermore, acupuncture reduces neuroinflammation through mast cell activation and inhibition of pro-inflammatory cytokines (IL-6, TNF-α) (21), regulates GABAergic signaling to restore basal ganglia balance (22), and suppresses microglial activation and oxidative stress via the TRPC1/Ca^2+^ pathway (23). Collectively, these findings suggest that acupuncture exerts multifaceted neuroprotective effects—through dopaminergic, neurotrophic, and neuroimmune modulation, to preserve motor function and potentially delay progression in early-stage PD.

Despite these promising findings, high-quality evidence specifically targeting early-stage PD remains limited. The majority of studies have focused on patients in the mid-to-late stages of PD (14, 24, 25), leaving a gap in evidence for early acupuncture intervention. Given the potential benefits of initiating adjunctive therapy during the early stage of PD and the absence of high-quality randomized controlled trials (RCTs) focusing on motor dysfunction in this population, we designed a single-blind, sham-controlled RCT to evaluate the efficacy and safety of standardized acupuncture in improving motor performance in early-stage PD. A 12-month follow-up was incorporated to assess the sustainability of treatment effects and provide further insights into acupuncture’s role in long-term clinical decision-making.

This study aimed to evaluate the efficacy and safety of acupuncture in improving motor function in early-stage PD. The results will help determine its potential role as an adjunctive therapy to preserve motor function.

## Methods and analysis

### Study design

**Figure 1:**
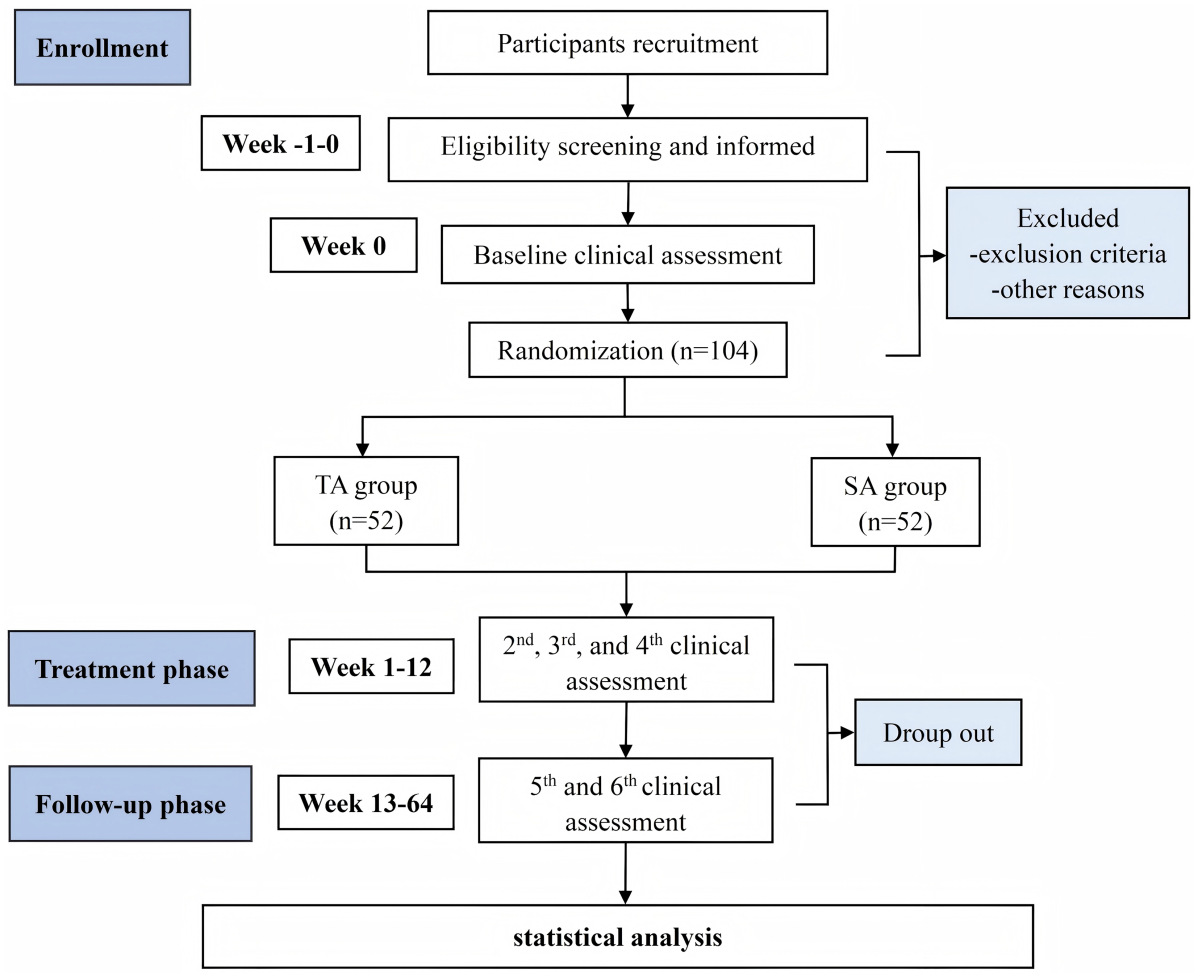
Flowchart of the study design.

This is a single-blind, randomized, sham-controlled clinical trial designed to evaluate the efficacy and safety of acupuncture in treating motor dysfunction in early-stage PD. The study will be conducted at two clinical sites in Chengdu, China: the Hospital of Chengdu University of Traditional Chinese Medicine and the First People’s Hospital of Longquan District. Both institutions have well-established neurology and TCM departments, ensuring the standardized implementation of the protocol and reliable participant recruitment. The trial is scheduled to run from 21 January 2025 to 31 December 2028. Participant recruitment and interventions will take place between 1 September2025 and 1 September 2027. A detailed study flowchart is presented in Figure 1. The protocol follows the Standard Protocol Items: Recommendations for Interventional Trials (SPIRIT) 2013 Statement guidelines for clinical trial protocols (see Supplementary file 1) and is conducted in accordance with the ethical principles outlined in the Declaration of Helsinki (26). Ethical approval has been granted by the Medical Ethics Committee of the Hospital of Chengdu University of TCM (Approval No. 2025KL-020). The trial will be reported in compliance with the guidelines of the Consolidated Standards of Reporting Trials (CONSORT) (27), and it has been registered with the International Traditional Medicine Clinical Trial Registry (No. ITMCTR2025001290). A detailed schedule of enrollment, interventions, and assessments is provided in Table 1.

**Table 1:** Study schedule for data measurements.

Assessment	Study period						
Time point	Enrolment	Baseline	Treatment phase (3 months)			Follow-up (1 year)	
	Week						
	−1	0	4	8	12	38	64
Enrolment							
Eligibility screen	**×**						
Informed consent	**×**						
Randomization		**×**					
Interventions							
TA group (*n* = 52)			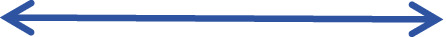				
SA group (*n* = 52)			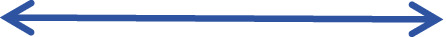				
Assessments							
CCMS		**×**	**×**	**×**	**×**	**×**	**×**
MDS-UPDRS		**×**	**×**	**×**	**×**	**×**	**×**
PPTS		**×**	**×**	**×**	**×**	**×**	**×**
TUG		**×**	**×**	**×**	**×**	**×**	**×**
6MWT		**×**	**×**	**×**	**×**	**×**	**×**
NMSS		**×**	**×**	**×**	**×**	**×**	**×**
PDQ-39		**×**	**×**	**×**	**×**	**×**	**×**
CGI		**×**	**×**	**×**	**×**	**×**	**×**
LEDD		**×**	**×**	**×**	**×**	**×**	**×**
Blinding assessment					**×**		
Treatment satisfaction					**×**		
Participants safety							
AEs			**×**	**×**	**×**	**×**	**×**
Laboratory test*		**×**			**×**		**×**

*The laboratory test includes complete blood count and differential count, absolute neutrophil count, aspartate aminotransferase, alanine aminotransferase, total bilirubin, blood urea nitrogen, creatinine, albumin, coagulation test, and 12-lead electrocardiography.

TA, true acupuncture; SA, sham acupuncture; CCMS, composite clinical motor score; MDS-UPDRS, MDS unified Parkinson’s disease rating scale; PPTS, Purdue Pegboard Test score; TUG, Timed Up and Go Test; 6MWT, 6-min walk test; NMSS, Non-Motor Symptoms Scale; PDQ-39, 39-item Parkinson’s Disease Questionnaire; CGI, Clinical Global Impression Scale; LEDD, levodopa equivalent daily dose; AEs, adverse events.

### Randomization and blinding

Participants will be randomly assigned in a 1:1 ratio to either the true acupuncture (TA) group or the sham acupuncture (SA) group using stratified block randomization by study center, with a fixed block size of four. The randomization sequence will be generated by an independent statistician using SPSS version 27.0 (IBM Corp., Armonk, NY, USA), with a predetermined random seed (seed = 20,250,504) to ensure reproducibility. Group allocations will be concealed in sequentially numbered, opaque, sealed envelopes. These envelopes will be opened by designated study coordinators only after participants have been formally enrolled.

Due to the nature of acupuncture, acupuncturists cannot be blinded. However, participants, outcome assessors, and data analysts will remain blinded throughout the study. To ensure allocation concealment and prevent potential unblinding, all treatment sessions will be conducted in separate rooms. Acupuncturists will receive standardized protocol training to minimize performance bias and to avoid any inadvertent disclosure of group assignments.

### Recruitment and informed consent

A total of 102 participants will be recruited from the neurology outpatient departments of the two participating centers. Recruitment strategies will include referrals from neurologists, announcements on hospital bulletin boards, official hospital websites, and posts via the WeChat platform. Interested individuals may contact the study coordinator by telephone, email, or in person. Initial eligibility screening will be conducted by trained neurologists, followed by detailed explanations of the study procedures. Written informed consent will be obtained from all participants prior to enrollment (see Supplementary file 2).

### Participants

#### Inclusion criteria

Participants will be eligible for inclusion if they meet all of the following criteria:

1. diagnosed with idiopathic PD according to the Movement Disorder Society Clinical Diagnostic Criteria for PD (2015) (28), with stable physical condition;
2. male or female, aged 40 and 80 years;
3. Hoehn and Yahr stage 1.0 to 2.5, meeting the definition of early-stage PD (29);
4. disease duration ≤3 years (30, 31), with a stable dosage of antiparkinsonian medications for at least 3 months before enrollment and expected to remain stable throughout the intervention and follow-up period (minimum 6 months);
5. voluntary participation with signed informed consent;
6. demonstrates good compliance and can complete the 12-week intervention and 1-year follow-up, including all planned study visits.

#### Exclusion criteria

Participants will be excluded if they meet any of the following criteria:

1. atypical or secondary parkinsonism, or any serious cardiovascular, cerebrovascular, hepatic, renal (creatinine clearance < 30 mL/min), hematologic, oncologic, or other neurological disorders (e.g., stroke and epilepsy);
2. diagnosis of severe psychiatric disorders (e.g., schizophrenia and major depressive disorder), or evidence of cognitive, hearing, or visual impairment that may interfere with assessments (MoCA < 26, ranging from 0 to 30, with a score of 26 or higher indicating normal cognitive function) (32);
3. untreated or uncontrolled physical pain that may affect gait, such as severe osteoarthritis or lumbar disc herniation;
4. use of medications affecting motor or neurological function (e.g., antipsychotics, anticholinergics, and benzodiazepines) within 30 days prior to enrollment, except for stable-dose antiparkinsonian drugs;
5. participation in another clinical trial within 30 days before screening;
6. presence of skin diseases affecting acupuncture sites, such as psoriasis or local infections;
7. history of allergic reactions to acupuncture or severe needle phobia.

Participants may withdraw at any time due to treatment intolerance, adverse events (AEs), or personal preference. Participants who experience serious AEs or become non-compliant will be advised to discontinue, and the reason and timing will be documented in the case report forms (CRFs). Outcome assessments will be sought from withdrawn participants whenever possible.

### Intervention

#### TA group

**Figure 2:**
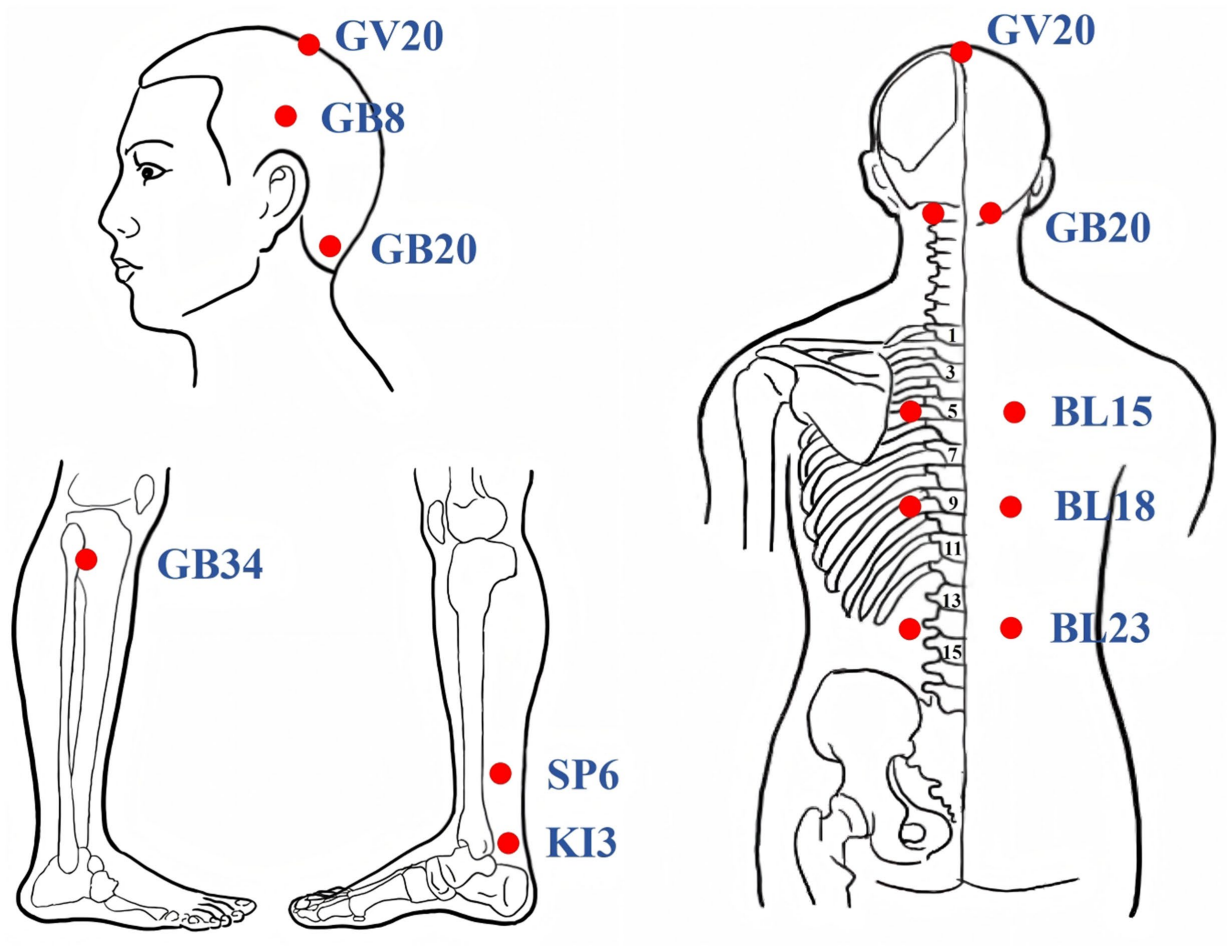
Location of acupoints.

Participants assigned to the TA group will receive manual acupuncture using sterile disposable stainless steel needles (0.25 mm × 40 mm, Huatuo, Suzhou, China). Acupoints were selected based on prior clinical and preclinical evidence (14, 15). Acupoint prescriptions included GV20 (Baihui), GB8 (Shuaigu), GB20 (Fengchi), GB34 (Yanglingquan), BL15 (Xinshu), BL18 (Ganshu), BL23 (Shenshu), SP6 (Sanyinjiao), and KI3 (Taixi). The acupoint locations adhere to the National Standard of the People’s Republic of China (GB/T1236-2021) (33), as shown in Table 2 and Figure 2. Needle insertion depth ranges from 10 to 30 mm, depending on anatomical site. Needles will be retained for 30 min each session, with manual stimulation (twisting and lifting-thrusting) performed every 15 min to elicit the “deqi” sensation (such as soreness, heaviness, or distension). Treatments will be administered three times weekly for 12 consecutive weeks, totaling 36 sessions. Patients will be positioned prone with eyes covered to maintain blinding. Skin will be disinfected prior to needling. The intervention protocol follows the Standards for Reporting Interventions in Clinical Trials of Acupuncture (34). Licensed acupuncturists (with ≥5 years of experience) will receive standardized training on protocol adherence and confidentiality, with performance monitored via treatment logs.

**Table 2:** Location of acupoints.

Acupoints	Location
GV20 (Baihui)	On the head, 5 cun superior to the fronterior hairline, on the fronterior median line.
GB8 (Shuaigu)	On the head, directly superior to the auricular apex, 1.5 cun superior to the temporal hairline.
GB20 (Fengchi)	In the anterior region of the neck, inferior to the occipital bone, in the depression between the origins of the sternocleidomastoid and the trapezius muscles.
GB34 (Yanglingquan)	On the fibular aspect of the leg, in the depression anterior and distal to the head of the fibula.
BL15 (Xinshu)	On the back, below the spinous process of the 5th thoracic vertebra, 1.5 cun aside the posterior median line.
BL18 (Ganshu)	On the back, below the spinous process of the 9th thoracic vertebra, 1.5 cun aside the posterior median line.
BL23 (Shenshu)	In the lumbar region, below the spinous process of the 2nd lumbar vertebra, 1.5 cun aside the posterior median line.
SP6 (Sanyinjiao)	On the medial side of the lower leg, 3 cun above the tip of the inner ankle, at the posterior margin of the medial border of the tibia.
KI3 (Taixi)	On the posteromedial aspect of the ankle, in the depression between the prominence of the medial malleolus and the calcaneal tendon

#### SA group

**Figure 3:**
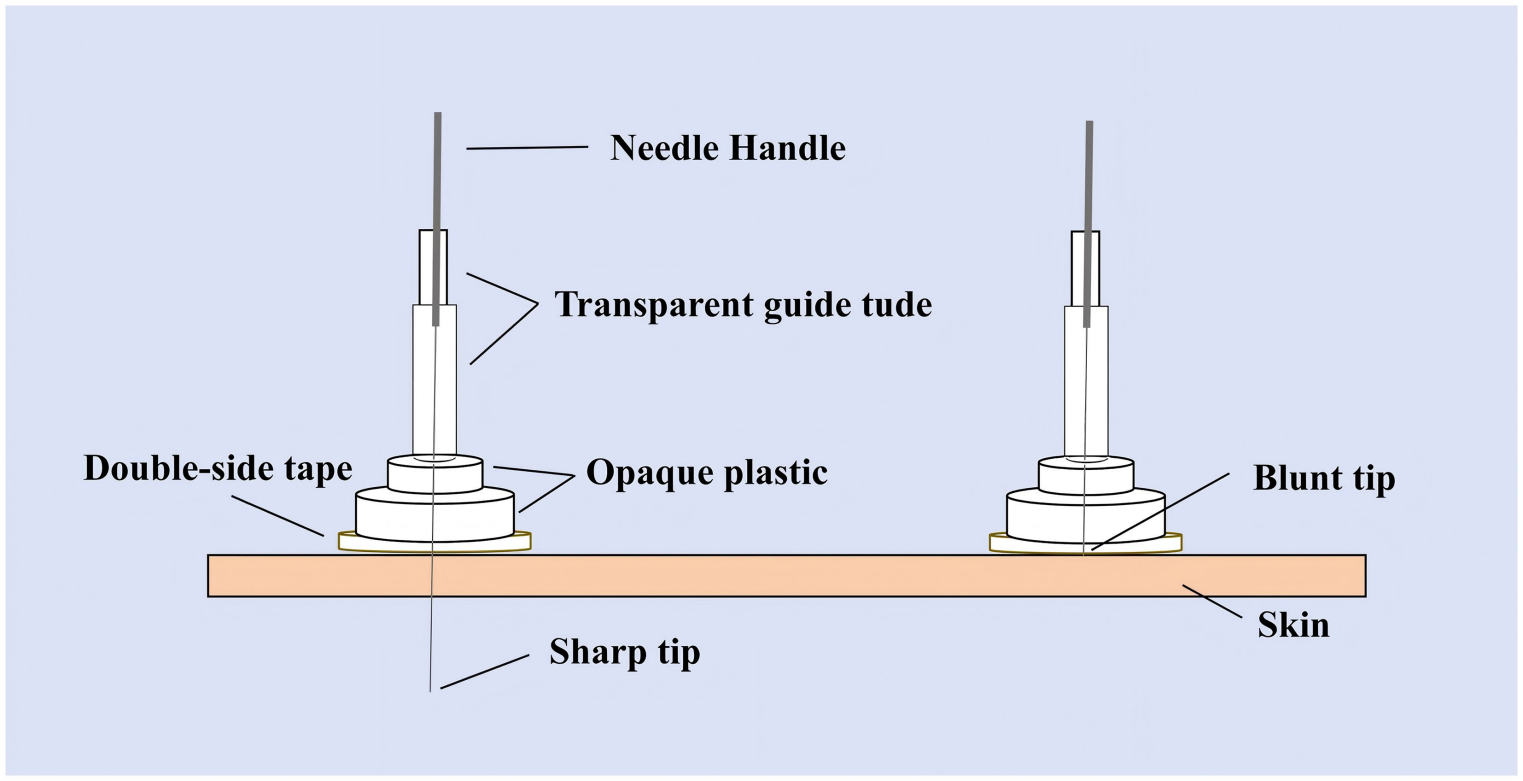
Park sham acupuncture device.

Participants in the SA group will receive treatment with the Park sham device applied to identical acupoints to ensure participant blinding. The device uses blunt, non-penetrating needles (0.30 × 25 mm and 40 mm, Huatuo, Suzhou, China) that simulate the sensation of needling without skin penetration (see Figure 3). The system comprises transparent tubes (Φ4 × 20 mm, Φ3 × 35 mm), double-sided adhesive tape (Φ1 × 15 mm), and opaque plastic bases (Φ4 × 15 mm, Φ5 × 10 mm), manufactured by Suzhou Medical Materials Factory, batch number 240407. Patients perceive needle contact without actual skin penetration. For scalp points, the blunt needle is secured with hairpieces to enhance stability (35). Treatment frequency, duration, and practitioner interaction mirror the TA group to maintain procedural consistency.

### Concomitant treatments

Participants who have been on stable antiparkinsonian medication regimens (dopamine agonists, levodopa, or MAO-B inhibitors, alone or in combination) for at least 3 months prior to enrollment will maintain their medication throughout the 12-week intervention and 1-year follow-up unless clinical indications necessitate adjustments (36). The use of additional antiparkinsonian or central nervous system agents is prohibited; any deviations will be documented and accounted for in statistical analysis. Medications for comorbid conditions (e.g., hypertension and diabetes) are permitted and will be documented at each visit. In case of symptom worsening, medication adjustments may be made under physician guidance, with changes recorded in CRFs and converted to levodopa equivalent daily dose (LEDD) for analysis. Stable non-acupuncture interventions (e.g., rehabilitation or exercise) unrelated to motor function are permitted; their use will be recorded at each visit and adjusted as covariates in the mixed-effects model analysis.

### Outcomes

#### Primary outcome

The primary efficacy endpoint is the change in the composite clinical motor score (CCMS) from baseline to week 12, assessed in the on-medication state. The CCMS is a validated composite index that integrates three key motor domains—bradykinesia (derived from MDS-UPDRS Part III, 40% weight), manual dexterity (from the Purdue Pegboard Test, 30% weight), and mobility (from the Timed Up and Go [TUG] Test, 30% weight)—to offer a sensitive, multidimensional evaluation of motor function. It is calculated as follows: [(MDS-UPDRS III − 23.8829) × 0.0377 + (−1 × Purdue Pegboard Test + 28.9286) × 0.0739 + (Timed Up and Go Test −9.8746) × 0.1414 + 3.8239] × 6.0946, providing a comprehensive assessment of motor function improvement, including bradykinesia, dexterity, and mobility (37). Developed and validated in the Oxford Discovery cohort of over 900 early-stage PD patients (37), CCMS outperforms MDS-UPDRS Part III alone, with a wider score distribution for subtle changes, a lower coefficient of variation (37% vs. 67%), and a stronger correlation with patient-reported motor severity (*r* = 0.65 vs. 0.61). Its enhanced longitudinal sensitivity in early-stage PD reduces sample size requirements by 64% in trial simulations, making it ideal for detecting acupuncture’s modest, clinically meaningful effects that conventional scales might overlook. Additionally, participants will undergo assessment in the off-medication state (after a 12-h withdrawal from antiparkinsonian medication) at week 12 to evaluate the independent effects of acupuncture on motor function.

#### Secondary outcomes

The secondary outcomes include change in CCMS from baseline to week 64. MDS unified Parkinson’s disease rating scale (MDS-UPDRS) (38) scores comprised Part I (non-motor aspects of daily living, range 0–52, higher scores indicate greater impairment), Part II (motor aspects of daily living, range 0–52, higher scores indicate greater impairment), Part III (motor examination), Part IV (motor complications), and total score (sum of Parts I–III, range 0–236, higher scores indicate greater impairment). Purdue Pegboard Test score (PPTS) assesses fine motor skills, with two trials averaged per session. The Timed Up and Go Test (TUG) measures the time to rise, walk 3 m, and return, with two trials averaged, and records the use of assistive devices. Six-minute walk test (6MWT) (39) measures the maximum distance walked in 6 min on a flat surface, with two trials averaged, and records the use of assistive devices, which will be conducted with researcher supervision for safety. The Non-Motor Symptoms Scale (NMSS) (15), ranging from 0 to 360, evaluates domains such as sleep/fatigue, mood, and gastrointestinal function. The 39-item Parkinson’s Disease Questionnaire (PDQ-39) (40), ranging from 0 to 100, covering 8 dimensions (e.g., mobility), measures health-related quality of life. Clinical Global Impression Scale (CGI), including CGI-S (severity) and CGI-I (improvement), will be completed by a blinded assessor. LEDD will be calculated according to the standardized conversion formula proposed by Tomlinson et al. (41), recording changes from baseline at each follow-up. All secondary outcomes will be assessed at baseline; at weeks 4, 8, and 12 (during treatment); and at weeks 38 and 64 (follow-up). Among these, the NMSS, as a key secondary outcome, will be analyzed using mixed-effects models to evaluate changes in sleep/fatigue and mood domains from baseline to month 15.

#### Other outcomes

Blinding Assessment: Participants will complete a questionnaire at weeks 12 and 64 asking, “Do you believe you received true acupuncture or sham acupuncture?” with options: “yes,” “no,” or “unsure” (see Supplementary file 3). The Bang Blinding Index will be calculated to evaluate the success of blinding. This will be performed by blinded assessors unaware of group allocation (24). Treatment satisfaction: AEs, such as local pain, bruising, dizziness, or infection, will be documented at every treatment visit. Routine laboratory tests (complete blood count and liver and renal function) will be performed at baseline, week 12, and week 64 to monitor safety (15).

#### Sample size calculation

As no prior acupuncture studies have reported data on the CCMS, the sample size estimation was based on a randomized controlled trial of acupuncture in PD, which reported a mean change in MDS-UPDRS Part III of −2.95 ± 0.94 in the treatment group and 3.96 ± 0.94 in the control group, yielding a between-group difference (*δ*) of −6.91 and a pooled standard deviation (*σ*) of 8.56 (Cohen’s *d* = 0.81) (15). Given that the CCMS incorporates MDS-UPDRS III (40% weight) and has demonstrated higher sensitivity and lower measurement variability compared with MDS-UPDRS III alone (37), the overall detectable effect size was expected to remain comparable, even considering potential attenuation due to the use of the Park sham needle. Therefore, *δ* = −6.91 and *σ* = 8.56 were retained for sample size estimation (Cohen’s *d* = 0.81), representing a realistic yet conservative assumption for identifying clinically meaningful motor improvement in early-stage PD. Using PASS version 21.0 (NCSS LLC, Kaysville, UT, USA) for a two-sample *t*-test, with a two-sided significance level (*α*) of 0.05 and statistical power (1 − *β*) of 90%, a total of 34 participants per group was required. Considering a 20% dropout rate, common in long-term PD trials, the adjusted sample size is 43 per group. To account for inter-center variability across the two study sites, the sample size was increased by 20% (42), resulting in 52 participants per group, totaling 104 participants.

### Statistical analysis

All statistical analyses will be conducted using SPSS version 28.0 (SPSS Inc., Chicago, IL, USA). Continuous variables will be presented as means ± standard deviations (for normally distributed data) or medians with interquartile ranges (for non-normally distributed data), determined using the Shapiro–Wilk test. Categorical variables will be expressed as counts and percentages. Between-group comparisons at baseline will be performed using independent-samples *t*-tests or Mann–Whitney *U* tests for continuous variables and chi-square tests or Fisher’s exact tests for categorical variables. Baseline covariates (e.g., age, sex, disease duration, and LEDD) will be adjusted where applicable. Longitudinal changes in primary and secondary outcomes will be analyzed using a mixed-effects model for repeated measures, with fixed effects for group, time, and group-by-time interaction, and adjusting for relevant covariates. In cases where the assumption of sphericity is violated (Mauchly’s test *p* < 0.05), the Greenhouse–Geisser correction will be applied. Post-hoc multiple comparisons will be adjusted using the Bonferroni method; sensitivity analyses will also be conducted using either unadjusted comparisons or the false discovery rate (FDR) approach. Primary analyses will follow the intention-to-treat (ITT) principle, including all randomized participants. Missing data will be handled using multiple imputation by chained equations (MICE) (24, 43), incorporating covariates (e.g., age and LEDD); sensitivity analyses will assess robustness for the 15-month follow-up. For blinding assessment, questionnaires will be administered at months 3, 12, and 15 to calculate the Bang Blinding Index, with post-hoc sensitivity analyses addressing potential biases. A per-protocol (PP) analysis will be performed on participants who complete ≥80% of scheduled treatment sessions. Exploratory subgroup analyses will examine acupuncture effects by gender, age (<60 vs. ≥60 years), and disease duration (≤1 vs. >1 year), using interaction terms in mixed-effects models to assess heterogeneity, interpreted cautiously due to power limitations. A two-tailed *p* value of < 0.05 will be considered statistically significant.

### Safety assessments

All participants will undergo routine safety evaluations at baseline and week 12, including complete blood count, liver and renal function tests, and 12-lead electrocardiography. AEs related to acupuncture (e.g., local pain, bleeding, bruising, dizziness, or transient fatigue) and medication use (e.g., nausea and gastrointestinal discomfort) will be systematically monitored and recorded at each study visit. AEs will be classified by severity using the Common Terminology Criteria for Adverse Events (CTCAE) v5.0 (mild grade 1, moderate grade 2, and severe grade ≥3). All AEs will be documented in a standardized CRF and reviewed by the study safety committee. Serious adverse events (SAEs), including hospitalization, permanent disability, or life-threatening conditions, will be reported to the institutional ethics committee within 24 h and followed until resolution. To ensure participant safety, individuals experiencing discomfort or side effects will receive immediate medical evaluation and care. Those facing adherence difficulties or treatment anxiety will be offered counseling and flexible scheduling. If unacceptable safety risks or unexpected SAEs emerge, the study will be suspended or terminated pending ethics committee review.

### Data management and quality control

All research personnel will undergo standardized training on protocol procedures, including participant screening, informed consent acquisition, CRF completion, and outcome assessments. Paper-based documents will be securely stored in locked filing cabinets, while electronic data will be maintained in encrypted, password-protected databases. Data monitoring will be conducted by the Clinical Research Center of Chengdu University of Traditional Chinese Medicine. An independent data monitoring committee will regularly review accumulated safety data, assess protocol adherence, and make recommendations regarding study continuation. Routine audits may be conducted by the ethics committee to ensure regulatory compliance. All records will be archived for a minimum of 5 years following study publication. Any protocol deviations or violations will be documented and reported to the ethics committee for evaluation and corrective action. To promote adherence, participants will use a digital app (e.g., WeChat-based reminders) for appointment tracking, with >80% completion required for per-protocol analysis; non-compliance will be mitigated through flexible scheduling and transportation support. For follow-up assessments, monthly phone reminders and incentives (e.g., travel reimbursement) will reduce attrition, with core outcomes (CCMS and PDQ-39) evaluated at all time points (baseline to month 15). Support includes transportation reimbursement, flexible scheduling, and a dedicated coordinator to address side effects or adherence issues, ensuring ethical transparency and participant wellbeing. AEs will be monitored at all visits (baseline to month 15), with delayed events (e.g., post-treatment fatigue) tracked via monthly phone follow-ups. SAEs will be reported to the ethics committee within 24 h for comprehensive long-term safety assessment.

### Trial status

The trial commenced in January 2025 and is currently at the stage of recruiting patients.

## Discussion

Early-stage PD, characterized by mild neurodegenerative changes, presents a critical window for intervention to slow disease progression, reduce reliance on levodopa, and enhance QoL (6). Although current first-line pharmacotherapies—such as levodopa and MAO-B inhibitors—are effective in alleviating motor symptoms, their long-term use is associated with complications including motor fluctuations, “on–off” phenomena, and levodopa-induced dyskinesia. Moreover, these treatments offer limited efficacy for NMS, such as anxiety and sleep disturbances (10, 11, 44). In China, the high cost of imported medications imposes a significant financial burden on patients. The bidirectional exacerbation of motor and NMS further compromises QoL (45). This study aimed to evaluate the clinical efficacy and safety of acupuncture for motor dysfunction in early-stage PD through a randomized, single-blind, sham-controlled trial, with 1-year follow-up data intended to support its integration into early care as a means to preserve motor function and reduce pharmacological burden.

The pathophysiology of PD involves complex mechanisms, including activation of the NLRP3 inflammasome, oxidative stress, and dysregulation of the gut–brain axis—all contributing to the degeneration of dopaminergic neurons (46, 47). Acupuncture has been shown to exert neuroprotective effects by inhibiting NLRP3 inflammasome activation, reducing oxidative stress, and upregulating brain-derived neurotrophic factor (48, 49). Furthermore, acupuncture modulates gut microbiota composition, thereby restoring gut–brain axis homeostasis (50). It also regulates immune balance by promoting Th17/Treg differentiation and inhibiting the TLR4/NF-κB signaling pathway, leading to reduced levels of pro-inflammatory cytokines such as IL-6 and TNF-*α* (51, 52). Clinically, randomized controlled trials and meta-analyses have reported improvements in tremor control, gait, and balance following acupuncture, as well as enhanced overall motor performance (14, 15). These multimodal effects align well with the multifactorial pathology of PD and support further investigation in early-stage patients.

This study targets patients with early-stage PD and uses a rigorously designed randomized, single-blind, sham-controlled trial. The use of the Park Sham needle ensures effective participant blinding, thereby minimizing placebo effects. Unlike levodopa, which risks dyskinesia in >50% of early-stage PD patients within 5–10 years (53), acupuncture offers a low-cost, tolerable adjunct without such complications, positioning it within the broader early-stage PD landscape. The acupuncture protocol in this study draws from TCM theory and modern neurophysiological evidence. In TCM, early-stage PD manifests as liver–kidney deficiency and qi–blood stagnation, impairing meridian nourishment and motor coordination. The treatment principle thus emphasizes nourishing the liver and kidney, invigorating qi and blood, and unblocking meridians to restore function. Complex network analysis of 326 clinical studies identified GV20, GB20, GB34, and SP6 as core acupoints for PD, combining Governor Vessel points to calm the mind with limb acupoints that regulate liver–kidney balance and dispel pathogenic wind (54). GV20 and GB20 modulate excitatory–inhibitory neurotransmission: electroacupuncture at these points suppresses striatal glutamate and acetylcholine release in parkinsonian rats, improving rotational behavior (55). GB34, a key motor acupoint, activates the precentral gyrus and prefrontal cortex in PD patients, enhancing synaptic dopamine availability and upregulating dopamine transporter/D1 receptor expression while normalizing D2 levels in 6-OHDA models (56). Collectively, these acupoints support a dual rationale—meridian regulation per TCM and targeted modulation of dopamine/glutamate signaling and inflammation—positioning acupuncture as a neuroprotective adjunct for early-stage PD motor dysfunction.

The treatment frequency of three sessions per week over 12 weeks optimizes therapeutic benefit while ensuring feasibility and adherence. To sensitively capture motor changes in early-stage PD, the CCMS (37) was selected as the primary outcome, integrating MDS-UPDRS Part III, PPT, and TUG for a multidimensional assessment of motor performance, surpassing traditional single-domain measures. The secondary outcomes, such as MDS-UPDRS and PPT, are validated for early-stage PD sensitivity: MDS-UPDRS exhibits high test–retest reliability (ICC > 0.9) in early-stage PD cohorts (57), enabling detection of subtle motor impairments, while the PPT is responsive to fine motor changes (58), capturing dexterity deficits common in early disease. These measures, alongside the 12-month follow-up, will evaluate motor benefit durability and potential pharmacological burden reduction.

This trial has several strengths. The use of CCMS as the primary outcome ensures high sensitivity and comprehensive assessment of motor function, surpassing traditional single-metric measures (37). The 12-week treatment period combined with a 12-month follow-up enables observation of both short- and long-term effects. The use of the Park sham device ensures robust participant blinding, reducing expectancy and placebo bias. Furthermore, the acupuncture protocol is standardized, based on clinical evidence and TCM theory, and follows national standards for acupoint localization. Stratified randomization and consistent intervention frequency (three sessions per week for 12 weeks) enhance clinical feasibility, adherence, and replicability.

The limitations of this study are as follows. First, differences in sensory perception (e.g., the sensation of “deqi”) may compromise blinding integrity (59); to mitigate this, blinding assessments are conducted at weeks 12 and 64, with post-hoc sensitivity analyses excluding suspected unblinded cases. Second, due to the nature of acupuncture, blinding of practitioners is not feasible, which may introduce performance bias. This risk will be minimized through standardized training and strict protocol adherence. Third, adherence may be a challenge for some patients due to the intensity of the treatment schedule (36 sessions in 12 weeks). To address this, the study team will offer transportation assistance, flexible appointment scheduling, and digital attendance tracking (e.g., WeChat check-ins) to support compliance. Finally, to ensure data homogeneity, participants with severe comorbidities or marked cognitive impairment are excluded, which may limit generalizability; future studies could expand inclusion criteria and extend follow-up durations. Additionally, while this trial compares acupuncture to sham, it does not directly contrast with standard pharmacological therapies such as levodopa for early-stage PD. Subsequent research should evaluate acupuncture’s head-to-head potential against drugs to further elucidate its role in early-stage management.

In conclusion, this study uses a rigorously designed randomized, single-blind, sham-controlled trial to evaluate the efficacy and safety of acupuncture for motor dysfunction in early-stage PD. By targeting a well-defined population, using a sensitive composite motor outcome, and standardizing the intervention, it aims to clarify the potential role of acupuncture as an adjunctive therapy to preserve motor function and reduce reliance on pharmacological treatment in the early-stage PD. Positive findings would provide a basis for integrating acupuncture into evidence-based early-stage PD care.
