# Supplementary material for: Efficacy of acupuncture for motor dysfunction in early Parkinson’s disease: protocol for a randomized, single-blind, sham-controlled clinical trial
Source: Front Med (Lausanne). 2025 Nov 19;12:1699907. doi: 10.3389/fmed.2025.1699907 (PMC12673886; doi:10.3389/fmed.2025.1699907)
Supplement: Supplementary file 1 [file Supplementary_file_1.docx]

**Informed consent**

**Clinical research project title:** Clinical Study on the Efficacy and Safety of Acupuncture for Motor Dysfunction in Early-Stage Parkinson’s Disease

**Project source:** Youth Fund Project of Sichuan Natural Science Foundation

**Name of Research Centre:** College of Acupuncture and Tuina, Chengdu University of Traditional Chinese Medicine, Kunming, China

**Name of the person in charge of this research project:** Fanrong Liang

**----------------------------------------------------------------------------**

Dear patient:

You have been diagnosed with Parkinson’s disease (PD), and we sincerely invite you to participate in a clinical research study entitled “Clinical Study on the Efficacy and Safety of Acupuncture for Motor Dysfunction in Early-Stage Parkinson’s Disease”
Please note that your participation in this study is entirely voluntary. The treatment you receive in this study differs from standard clinical care and will follow the protocol approved for clinical research. Before you decide whether to participate, please read the following information carefully. This form explains the purpose of the study, the procedures and timeline involved, and the possible benefits, risks, and inconveniences of participation. You may discuss the study with your family, friends, or doctor to assist you in making an informed decision.

If you agree to participate, you will be asked to sign this informed consent form. A signed copy will be provided to you for your records. This project has been approved by the Administrative Office of the Sichuan Provincial Clinical Research Center for Acupuncture and the Medical Ethics Committee of the Affiliated Hospital of Chengdu University of Traditional Chinese Medicine.

**Introduction to the Study**

1. **Research Background and purpose**

Parkinson’s disease (PD) is a chronic, progressive neurodegenerative disorder and the second most common neurodegenerative disease globally. As of 2020, over 10 million individuals are affected worldwide, with projections reaching 17 million by 2040 due to aging populations. In China, PD imposes a significant burden, with an age-standardized prevalence of 245.7 cases per 100,000 people, and an estimated 5 million patients by 2030. Early-stage PD (Hoehn and Yahr ≤ 2.5) is characterized by mild but functionally significant motor symptoms such as tremor, bradykinesia, rigidity, and postural instability. These symptoms impair daily activities (e.g., walking, writing, dressing), reducing quality of life (QoL). Non-motor symptoms like anxiety, depression, sleep disturbances, and constipation often emerge even in early stages, worsening disability and emotional burden. These clinical features highlight the need for early and comprehensive intervention.

Pharmacotherapy (e.g., levodopa and MAO-B inhibitors) is the mainstay treatment in early PD. While levodopa is effective in improving motor symptoms, long-term use often leads to motor complications such as wearing-off and dyskinesias, affecting more than 50% of patients within 5–10 years. MAO-B inhibitors’ disease-modifying effects remain unproven and have limited benefits for non-motor symptoms. Additionally, adverse effects may impair tolerance and do not halt neurodegeneration. Therefore, there is a growing demand for safe, accessible adjunct therapies that can address both motor and non-motor symptoms and reduce long-term medication dependence.

Acupuncture, a hallmark therapy in traditional Chinese medicine (TCM), has shown potential as an adjunctive therapy for PD. Randomized controlled trials and meta-analyses suggest that acupuncture (manual, electroacupuncture, scalp acupuncture) improves motor function, evidenced by reductions in MDS-UPDRS Part III scores. Stimulation of specific acupoints (e.g., Yanglingquan, Zusanli, Fengfu) is associated with tremor control, balance, and gait improvement in early PD. Scalp acupuncture may enhance gait via modulation of frontal and cerebellar cortical activity. Acupuncture may also benefit sleep, anxiety, and QoL, offering a well-tolerated, holistic intervention.

However, current evidence for acupuncture in early PD remains limited, despite its promise in symptom relief and reduced dopaminergic dependence. Most existing studies focus on moderate-to-advanced PD, leaving a critical gap in early intervention research. Thus, this trial aims to evaluate the clinical efficacy and safety of acupuncture in early-stage PD via a randomized, single-blind, sham-controlled design. The results are expected to provide evidence for integrating acupuncture into early PD care, potentially improving symptoms and reducing medication reliance.

This study will be conducted from January 2025 to December 2027 at two sites: the Affiliated Hospital of Chengdu University of TCM and Longquanyi District First People's Hospital. A total of 104 participants will be enrolled.

.

**Ⅱ. Who will be invited to participate in the study?**

1. **Inclusion criteria**

(1) Diagnosed with idiopathic PD based on the 2015 MDS Clinical Diagnostic Criteria, with a stable condition;

(2) Aged 40–80 years, regardless of gender;

(3) Hoehn and Yahr stage 1.0–2.5 (early-stage PD);

(4) Disease duration ≤3 years; stable anti-Parkinsonian medication dose for ≥3 months prior to enrollment and expected to remain stable for ≥6 months;

(5) Voluntarily agree to participate and sign informed consent;

(6) Able to comply with the 12-week intervention and 1-year follow-up, including all visits and assessments.

(Note: Only patients who met the above 6 criteria were included in this study.)

**2. Exclusion criteria**

(1) Atypical/secondary parkinsonism, or serious comorbidities (e.g., heart, liver, kidney failure, cancer, stroke, epilepsy);

(2) Severe psychiatric disorders or cognitive impairment (MoCA < 26), hearing/visual impairment affecting assessment;

(3) Pain or conditions affecting gait (e.g., osteoarthritis, disc herniation);

(4) Recent use of CNS-affecting drugs (e.g., antipsychotics, benzodiazepines);

(5) Participated in another clinical trial within 30 days;

(6) Skin conditions affecting acupuncture sites;

(7) Allergies to acupuncture or severe needle phobia.

(Note: Patients with any one of these parameters were not included in this study.)

**Ⅲ. Who will be excluded from the trial?**

**1. Exclusion and drop-out criteria**

(1) if the patients were mistakenly included, such as concealment, and were found not to meet the inclusion criteria later;

(2) those who were not treated as prescribed or whose data were incomplete;

(3) Those who had poor compliance or withdrew from the study because they did not meet the inclusion criteria would be excluded;

(4) changing the intervention method without authorization during the study;

(5) those who voluntarily request to withdraw due to serious adverse reactions, special physiological changes, or other unexpected events during the study.

**2. Removal and shedding treatment**

(1) For the drop-out subjects, the reasons for the drop-out should be clarified as far as possible, and the recent trial data collection should be improved. There will be no recruitment for the drop-out subjects. Patients and their families are recommended to go to specialized hospitals for comprehensive treatment due to aggravation of disease or serious adverse reactions. If they go to affiliated hospitals and cooperative hospitals of our university, priority treatment will be given according to special needs.

(2) For those who had completed one observation cycle, the relevant data were included in the statistical processing within the cycle.

**Ⅳ. What will I need to do if I participate in the study?**

The study is expected to last for 3 months. If you are enrolled in the study, you will first need to undergo relevant examinations to verify that you meet all the requirements for participation in the study.

**1. Before you are enrolled in the study, you will undergo:**

(1) Medical history review and physical examination;

(2) ECG, blood, urine, and stool tests.

**2. If you meet the inclusion criteria, the following steps will be taken for the study**

(1) Receive stable levodopa-based therapy (unchanged from prior use);

(2) Be randomly assigned (1:1) to true acupuncture or sham acupuncture, determined by computer randomization (blinded allocation);

(3) Receive acupuncture 3 times/week for 12 weeks (30 minutes/session, manipulation every 15 minutes), followed by 1-year follow-up.

(4) You will be asked to: Complete assessments before and after treatment, including motor symptoms, non-motor symptoms, QoL, emotional state, and acupuncture experience.

**Ⅴ. Possible benefits of participating in the study**

You may experience symptom relief from acupuncture. This study may also contribute to better understanding of acupuncture’s clinical effects in early PD. Upon study completion, you will be eligible for free health consultation and three additional acupuncture sessions within the next 15 months.

**Ⅵ. Risks and Discomforts**

Possible risks include dizziness, local pain, bleeding, bruising, or infection. All adverse events (AEs), whether related to the study or not, will be documented and managed by medical staff. Details such as AE onset, duration, severity, treatment, and outcome will be recorded and preserved. Serious adverse events (SAEs) will be promptly reported to the ethics committee.

**Ⅶ. Possible adverse reactions, risks, discomfort, and inconvenience of participating in the study**

You may feel sore, numb, heavy, and swollen during acupuncture, which are normal reactions to acupuncture. There may be adverse reactions after acupuncture, but they are few and mild. During acupuncture, you may feel dizzy because of your physical problems or emotional stress, which can be relieved after stopping acupuncture and proper rest. There may be bleeding, hematoma, and other phenomena after acupuncture, which can disappear after local pressure. But if an infection develops at the site of the needle, your doctor will treat it promptly.

If you experience any discomfort, new changes in your condition, or any unexpected situation, whether related to medication or acupuncture treatment, during the study period, you should notify your doctor immediately, and he/she will make a judgment and give appropriate medical treatment.

During the study period, you need to visit the hospital on time for follow-up and some examinations, which may cause you trouble or inconvenience.

**Ⅷ. Alternative Treatment Options**

You may receive standard treatments for PD, including dopaminergic medications (e.g., levodopa, dopamine agonists, MAO-B inhibitors). Adjustments may be made by your neurologist if symptoms worsen. Acupuncture is optional and may not benefit all patients equally.

**Ⅷ. Confidentiality**

Your medical data will be securely stored. Only authorized personnel (researchers, ethics committee, sponsor representatives) will have access. Your identity will remain confidential in any publication. We will protect your privacy to the extent permitted by law.

**Ⅸ. Voluntary Participation and Right to Withdraw**

Participation is entirely voluntary. You may withdraw at any time without penalty or loss of benefits. Your medical care will not be affected. Researchers may also withdraw you from the study for your safety or other reasons. You may be asked to undergo final assessments before withdrawal.

**Ⅹ. How to get more information?**

If you have any questions, suggestions, or complaints about this study, please promptly discuss them with the research team. Contact information is available on the signature page. If you feel inconvenient to communicate with the research team, you can consult or complain to the Medical Ethics Committee of the Hospital of Chengdu University of Traditional Chinese Medicine. Ethics Committee contact number: 028-87783142.

**Informed consent · Consent signature page**

**Statement of Subjects**

1. I have read the above description of this study and had the opportunity to discuss and ask questions with doctors about this study. All my questions were satisfactorily answered.

2. I am aware of the possible risks and benefits of participating in this study. I acknowledge that participation in the study is voluntary, confirm that I have had ample time to consider it, and understand that:

I can always consult the doctor for more information.

(2) I can withdraw from this study at any time without discrimination or retaliation, and my medical treatment rights and interests will not be affected.

(3) I also know that if I withdraw from the study, especially if I withdraw from the study due to acupuncture or drug treatment, it will be very beneficial to the whole study if I inform the doctor of my condition changes and complete the corresponding physical examination and physical and chemical examination.

(4) If I need to take any other medication because of a change in my condition, I will seek advice from my doctor beforehand or tell my doctor afterward.

(5) I consent to the representatives of the Food and Drug Administration, ethics committee, or sponsor to access my research data.

I will be provided with a signed and dated copy of the informed consent form.

Finally, I decided to agree to participate in the study and promise to follow my doctor's advice to the best of my ability.

Patients with signature: _____________________ on ___________ _______ _________ years

Contact number:_____________________

I confirmed that the details of the study, including its rights and possible benefits and risks, were explained to the patient, and I gave her a copy of her signed informed consent.

The doctor signature: _____________________ on ___________ _______ _________

Contact number:_____________________

I have read this informed consent form carefully, I have had the opportunity to ask questions, and all questions have been answered. I understand that

Participation in the trial is voluntary, and I can choose not to participate in the trial or withdraw at any time with notice to the investigators without facing discrimination or retaliation. My medical treatment and rights will not be affected.

The investigators could terminate my participation in the trial if I needed additional diagnosis/treatment if I did not adhere to the trial plan, or if I had other reasonable reasons.

I have voluntarily agreed to participate in the trial, and I will receive a copy of the signed informed consent form.

Signature: the participants.. on _______ _____ __________

**Statement by the subject's family**

1. I have read the above description of this study and had the opportunity to discuss and ask questions with doctors about this study. All my questions were satisfactorily answered.

2. I am aware of the possible risks and benefits of participating in this study. I acknowledge that participation in the study is voluntary, confirm that I have had ample time to consider it, and understand that:

I can always consult the doctor for more information.

(2) Patients could withdraw from this study at any time without discrimination or retaliation, and their medical treatment rights and interests would not be affected.

(3) I also know that if a patient drops out of the study, especially due to acupuncture or drug treatment, it will be very beneficial for the whole study if I inform the doctor of the patient's condition changes and complete the corresponding physical examination and physical and chemical examination.

(4) If the patient needs to take any other medical treatment because of the change in condition, I will seek the doctor's advice in advance or tell the doctor the truth afterward.

(5) I agree that the representatives of the Food and Drug Administration, ethics committee, or sponsor will have access to the study data of the patient.

I will be provided with a signed and dated copy of the informed consent form.

Finally, I supported the patient's wishes and agreed to participate in the trial, pledging to follow her advice and to be with her throughout her care.

Subjects guardian (families) signature: __________________ on _____ _____ _____

Relations with the subjects:.., contact phone number: ___________________

Statement by the Investigators

I have accurately informed the content of the informed consent form and answered the questions of the subjects and their families, and the subjects volunteered to participate in this clinical trial.

The researchers signature: ________________ on _______ _____ _______

**知情同意书·知情告知页**

**临床研究项目名称：**针灸干预早期帕金森病运动功能障碍的临床疗效研究

**项目来源**： 四川省自然科学基金青年项目

**研究中心名称**：成都中医药大学针灸推拿学院

**本中心主要研究者姓名**：梁繁荣

—————————————————————————————————

亲爱的朋友：

医生已经确诊您患有帕金森，我们邀请您参加一项课题为“针灸干预早期帕金森病运动功能障碍的临床疗效研究”的研究。首先，您需要了解的是参与这项研究是完全自愿的。其次，在临床研究中接受的治疗与常规医疗存在很大差异，研究团队需要遵照临床研究方案的要求对您进行治疗。

在您决定是否参加这项研究之前，请尽可能仔细阅读以下内容。它可以帮助您了解该项研究以及为何要进行这项研究，研究的程序和期限，参加研究后可能给您带来的益处、风险和不适。如果您愿意，您也可以和您的亲属、朋友一起讨论，或者请医生给予解释，帮助您做出决定。如您选择参与这项临床研究，您需要签署这份知情同意书。同时，您将收到一份签署后文件的副本，以供保存。

该项目已由四川省针灸临床医学研究中心管理部门立项批准，成都中医药大学附属医院医学伦理委员会已经对其进行伦理审查并同意开展

**一、研究背景和研究目的**

帕金森病（PD）是一种慢性、进展性神经退行性疾病，是全球第二大常见的神经退行性疾病。截至2020年，全球已有超过1000万人受其影响，预计到2040年，受全球老龄化的影像，这一数字将上升至1700万。在中国，帕金森病造成了沉重的负担，其年龄标准化患病率为每10万人245.7例，预计到2030年，患者数量将达到500万。

早期帕金森病通常被定义为霍恩和亚尔（Hoehn and Yahr）分级≤2.5级，其特征为轻度但具有功能相关性的运动功能障碍，如震颤、运动迟缓、强直和姿势不稳。这些症状会干扰日常活动，包括行走、书写和穿衣，从而降低患者的生活质量。除了运动功能障碍外，许多患者即使在早期阶段也会经历非运动症状，如焦虑、抑郁、睡眠障碍和便秘，这些症状可能会进一步加重残疾和情感负担。这些临床特征凸显了对及时且全面干预措施的需求。

药物治疗仍然是帕金森病早期的首选主要治疗。左旋多巴以及单胺氧化酶-B（MAO-B）抑制剂（如司来吉兰和雷沙吉兰）常被用于缓解运动症状。然而，尽管左旋多巴在改善运动功能方面效果显著，但其长期使用常与运动并发症相关，包括“药效减退”现象和左旋多巴诱导的异动症，超过50%的患者在5至10年内会出现这些并发症。MAO-B抑制剂的疾病修饰作用尚未得到证实，其在改善非运动症状方面的效果也较为有限。此外，这些药物无法阻止神经退行性的进展，且由于不良反应，部分患者可能难以耐受。因此，对于安全、可及且能够同时解决帕金森病运动和非运动方面问题的辅助疗法的需求日益增加，这些疗法可能减少对药物治疗剂量递增的需求。

针灸是传统中医的特色疗法之一，目前已经作为一种有潜力的帕金森病辅助疗法被应用与临床。随机对照试验和荟萃分析表明，针灸方式（包括手法针灸、电针和头针）显著改善运动功能，这通过运动障碍协会统一帕金森病评分量表（MDS-UPDRS）第三部分评分的降低得到证实。刺激特定穴位，如胆经阳陵泉、胃经足三里和督脉风府，已被证明与早期帕金森病患者震颤控制、平衡和步态改善相关。此外，头针已被证明可以增加步长和行走速度，这可能是通过调节大脑中额叶和小脑等区域的皮层活动实现的。针灸还改善非运动症状，包括睡眠质量、焦虑和生活质量。针灸作为一种整体且耐受性良好的干预手段，在早期帕金森病的治疗中具有巨大的潜力。

然而，尽管目前已有不少研究支持针灸的疗效，但针对早期帕金森病的针灸研究仍显不足。早期阶段是干预的关键时期，非药物疗法有望减轻症状、延缓功能衰退，并减少对多巴胺类药物的长期依赖。目前的研究大多集中在中晚期帕金森病，导致早期帕金森病针灸研究的证据缺口较大。因此，有必要评估针灸作为早期帕金森病一线辅助疗法的效果。

本研究旨在通过随机、单盲、假针对照试验，评估针灸对早期帕金森病运动功能障碍的临床疗效和安全性。本研究的结果将为针灸整合到帕金森病早期护理中，特别是作为一种减少药物依赖和改善患者多方面症状的手段提供临床证据。

本试验将于2025年01月至2027年12月分别于成都中医药大学附属医院和龙泉驿区第一人民医院，预计将招募104位受试者参加。

**二、哪些人会被邀请参加这项研究？**

1.符合以下条件的人，将被邀请参加这项研究：

(1) 根据国际运动障碍学会《运动障碍临床诊断标准（2015）》被诊断为特发性帕金森病，身体状况稳定；

(2) 男女不限，年龄在40-80岁之间；

(3) Hoehn和Yahr分期1.0-2.5，符合早期 PD 的诊断；

(4) 病程≤3 年，入组前至少服用3个月稳定剂量的抗帕金森药物，并在整个干预和随访期间（至少6个月）保持稳定；

(5) 自愿参与并签署知情同意书；

(6) 表现出良好的依从性，能够完成为期12周的干预和为期1年的随访，包括所有计划中的研究访问。

注：同时具备以上6点者，方可纳入研究

2.如果您存在以下情况将不适宜参与本研究：

(1) 不典型或继发性帕金森病，或患有严重的心脑血管、肝、肾、造血系统疾病、恶性肿瘤或其他神经系统疾病（如卒中、癫痫）；

(2) 诊断出严重的精神障碍（如精神分裂症、重度抑郁症），或有可能干扰评估的认知、听力或视力障碍的证据；

(3) 可能影响步态的未治疗或未控制的身体疼痛，如严重的骨关节炎或腰椎间盘突出症；

(4) 入组前 30 天内服用过影响运动或神经功能的药物（如抗精神病药物、抗胆碱能药物、苯二氮卓类药物），但稳定剂量的抗帕金森药物除外；

(5) 在本研究所选用的穴位区域存在皮肤病、肢体残缺等状况而无法施行针灸治疗者；

(6) 对针刺治疗有过敏史或严重恐针症；

(7) 入组前30天内参加过其他临床试验。

注：符合上述任何一点，即予排除。

您的研究医生会对您进行检查，以确认您是否可以参加本研究。

**三、如果参加研究将需要做什么？**

1. 在您入选研究前，您将接受以下检查以确定您是否可以参加研究：

- 医生将询问、记录您的病史，并进行体格检查。
- 您需要做心电图、血常规、尿常规、大便常规。

2. 若您是筛查符合纳入标准的帕金森患者，将按以下步骤进行研究：

- 研究开始医生将根据您身体的具体情况提供左旋多巴的基础治疗，如在入组前已经服用药物，则不能随意更改剂量，换成等剂量的左旋多巴常规治疗。
- 医生首先将根据随机数字决定您接受何种治疗。您有1/2的可能性被分入真针刺组或假针刺组，您和您的医生都无法事先知道和选择分组。各组均是遵照临床常用针灸的绿色疗法。该研究针刺12周，每周3次，针刺时间持续30分钟，期间每15分钟行针一次。然后继续随访1年。故在参与前您需要确定您可以配合并完成干预和随访。
- 您接受的针刺治疗是安全无副作用的治疗措施，有利于缓解您帕金森引起的运动功能等症状，不会对您的健康造成损害。
- 针刺治疗期间您暂不能活动，针刺治疗周期后，遵医嘱予以常规药物治疗。
- 针刺治疗前后，会对您进行基本情况评估，包括运动症状、非运动症状、生活质量、情绪状况、针刺过程中的感受等，请您如实填写。

**四、参加研究可能的受益**

您将可能从本项研究中受益,。如果您愿意接受针刺治疗，您的病情将可能得到缓解。本项研究也有可能帮助医生和研究人员进一步确认针刺对帕金森的临床有效性研究，以用于患有相似病情的其他病人。如果您按计划完成试验，将在将来的15个月内获得项目组提供的免费健康咨询服务和3个疗程的针刺体验。

**五、参加研究可能的不良反应、风险和不适、不方便**

在针刺过程中可能会出现的晕针、针刺部位疼痛、出血、血肿或感染等。当发生不良事件时，无论其是否与本研究治疗方法有关，均应及时通知您的医生，他/她将对此做出判断并给与适当的医疗处理。并且，研究者会进行详细记录，包括：不良事件与严重不良事件的发生时间、中止时间和持续时间（可以用天或h来记录），严重程度及频率，处理方法及结果，对不良事件与试验治疗方法因果关系的分析，不良事件与严重不良事件的跟踪情况等。有关不良事件的所有临床资料，如检查单据、处方等均应会保存在原始文件中。

**六、除参加本研究外，您可以选择相应的治疗**

您的研究医生将与您讨论目前针对您的病情可选择的治疗方案，包括相应的风险和益处。针对帕金森的患者，目前可以选择药物治疗和非药物治疗，其中西医常规治疗包括在诊断后接受稳定的抗帕金森药物治疗（多巴胺受体激动剂、左旋多巴或单胺氧化酶 B [MAO-B]抑制剂，单独使用或联合使用），除有其他临床指征。禁止使用额外的抗帕金森药物或中枢神经系统药物；入症状加重，神经内科医生将根据您的症状进行用药调整，相关治疗将会被记录在CRF表中。针灸联合治疗可能缓解帕金森相关症状，也可能无法缓解帕金森症状，存在治疗的个体差异。

**七、有关费用**

您的针刺治疗的费用、血常规、大便常规及心电图的费用将由项目组承担。

如果在临床试验中出现不良事件，医学专家委员会将会鉴定其是否与针刺有关。如不良事件为针刺所引起，项目组将提供相应的经济补偿。对于您同时合并的其他疾病所需的治疗和检查，将不在免费的范围之内。在您完成试验后项目组将不再给予额外的受试者补偿费。

**八、个人信息是保密的吗？**

您的医疗记录（研究病历/CRF、化验单等）将完整地保存在您所就诊的医院。医生会将化验检查结果记录在您的病历上。研究者、申办者代表、伦理委员会和项目管理部门将被允许查阅您的医疗记录。任何有关本项研究结果的公开报告将不会披露您的个人身份信息。我们将在法律允许的范围内，尽一切努力保护您个人医疗资料的隐私。

**九、可以自愿选择参加研究和中途退出研究**

是否参加研究完全取决于您的意愿。您可以拒绝参加此项研究，或在研究过程中的任何时间退出本研究，这都不会影响您和医生间的关系，都不会影响对您的医疗或有其他方面利益的损失。

出于对您的最大利益考虑，医生或研究者可能会在研究过程中随时中止您继续参加本项研究。

如果您因为任何原因从研究中退出，您可能被询问有关您进行针刺治疗的情况。如果医生认为需要，您也可能被要求进行实验室检查和体格检查。

**十、怎样获得更多的信息？**

如您对这项研究存在任何疑问、建议或投诉，请及时与研究医生讨论，联系方式见签字页。如您感觉不便与课题组沟通，可向成都中医药大学附属医院医学伦理委员会进行咨询或投诉，联系电话：028-87783142。

**知情同意书·同意签字页**

**项目名称：**针灸干预早期帕金森病运动功能障碍的临床疗效研究

**同意声明**：

我已经阅读了上述有关本研究的介绍，而且有机会就此项研究与医生讨论并提出问题。我提出的所有问题都得到了满意的答复。我知道参加本研究可能产生的风险和受益。我知晓参加研究是自愿的，我确认已有充足时间对此进行考虑，而且明白：

- 我可以随时向医生咨询更多的信息。
- 我可以随时退出本研究，而不会受到歧视或报复，医疗待遇与权益不会受到影响。
- 我同样清楚，如果我中途退出研究时，我若将我的病情变化告诉医生，完成相应的体格检查和理化检查，这将对整个研究十分有利。
- 如果因病情变化我需要采取任何其他的药物治疗，我会在事先征求医生的意见，或在事后如实告诉医生。
- 我同意研究者、申办者代表、伦理委员会和项目管理部门查阅我的研究资料。
- 我将获得一份经过签名并注明日期的知情同意书副本。

最后，我决定同意参加本项研究，并保证尽量遵从医嘱。

患者签名： ＿ ＿ 年 ＿ ＿ 月 ＿ ＿ 日

联系电话（手机号码）：

我确认已向患者解释了本试验的详细情况，包括其权利以及可能的受益和风险，并给其一份签署过的知情同意书副本。

医生签名： ＿ ＿ 年 ＿ ＿ 月 ＿ ＿ 日

工作电话（手机号码）：
