# Supplementary material for: Efficacy of acupuncture for motor dysfunction in early Parkinson’s disease: protocol for a randomized, single-blind, sham-controlled clinical trial
Source: Front Med (Lausanne). 2025 Nov 19;12:1699907. doi: 10.3389/fmed.2025.1699907 (PMC12673886; doi:10.3389/fmed.2025.1699907)
Supplement: Supplementary file 2 [file Supplementary_file_2.docx]

**Blinding assessment**

| **Name: Random number: Date:** | |
| --- | --- |
| Please answer the following questions according to your latest acupuncture experience. | |
| 1. How does the needling sensation feel (sensation induced by needling, like soreness, numbness, swelling, heaviness, etc.)? | |
| Please choose a number between 0 (no needle sensation) and 10 (unbearable needle sensation). | ___________________ |
| 2. Have you ever received acupuncture? | Yes □ No □ |
| 3. Do you think you received true acupuncture or sham acupuncturet? | Yes □ No □  Unsure □ |
| How sure are you on your answer on a scale of 0 to 10? (0 = very uncertain and 10 = completely certain) | ___________________ |
